# Neurodegeneration and Neuroprotection in Diabetic Retinopathy

**DOI:** 10.3390/ijms14022559

**Published:** 2013-01-28

**Authors:** Mohammad Shamsul Ola, Mohd Imtiaz Nawaz, Haseeb A. Khan, Abdullah S. Alhomida

**Affiliations:** Department of Biochemistry, Faculty of Science, King Saud University, Riyadh 11415, Saudi Arabia; E-Mails: imtiaz.nawaz@gmail.com (M.I.N.); khan_haseeb@yahoo.com (H.A.K.); alhomida@ksu.edu.sa (A.S.A.)

**Keywords:** diabetic retinopathy, drugs, neurodegeneration, retina, oxidative stress, glutamate

## Abstract

Diabetic retinopathy is widely considered to be a neurovascular disease. This is in contrast to its previous identity as solely a vascular disease. Early in the disease progression of diabetes, the major cells in the neuronal component of the retina consist of retinal ganglion cells and glial cells, both of which have been found to be compromised. A number of retinal function tests also indicated a functional deficit in diabetic retina, which further supports dysfunction of neuronal cells. As an endocrinological disorder, diabetes alters metabolism both systemically and locally in several body organs, including the retina. A growing body of evidences indicates increased levels of excitotoxic metabolites, including glutamate, branched chain amino acids and homocysteine in cases of diabetic retinopathy. Also present, early in the disease, are decreased levels of folic acid and vitamin-B12, which are potential metabolites capable of damaging neurons. These altered levels of metabolites are found to activate several metabolic pathways, leading to increases in oxidative stress and decreases in the level of neurotrophic factors. As a consequence, they may damage retinal neurons in diabetic patients. In this review, we have discussed those potential excitotoxic metabolites and their implications in neuronal damage. Possible therapeutic targets to protect neurons are also discussed. However, further research is needed to understand the exact molecular mechanism of neurodegeneration so that effective neuroprotection strategies can be developed. By protecting retinal neurons early in diabetic retinopathy cases, damage of retinal vessels can be protected, thereby helping to ameliorate the progression of diabetic retinopathy, a leading cause of blindness worldwide.

## 1. Introduction

Diabetic retinopathy (DR) is a major complication of diabetes. It causes vision loss and blindness among adults worldwide. With the growing diabetes epidemic, the incidence of DR is increasing, along with the concomitant socio-economic burdens on families and the health system [1]. In recent years, many treatment options have been developed at late stages of the disease. These are often targeted at retinal vasculature and have been of limited benefit. However, to date, no drugs are available that prevent the incidence or progression of DR. In the past, diabetic retinopathy was considered solely a vascular disease; however, it is now widely recognized to be a neuro-vascular disease. Numerous early retinal function tests in diabetic retinopathy patients, as well as a number of cellular and molecular studies in the retinas of diabetic experimental animals, suggest that neurons are vulnerable to damage shortly after the onset of diabetes. This vulnerability exists before any sign of vascular damage [2,3]. Several clinical tools indicate functional deficits in the neuronal component of retinas during the early stages of diabetes [4,5]. These clinical tools include multifocal electroretinography (ERG), flash ERG, contrast sensitivity, color vision and short-wavelength automated perimetry. We and others have reported that diabetes causes a chronic loss of retinal neurons by increasing the frequency of apoptosis and the activation of glial cells [2,3,6,7] (Ola *et al.* unpublished data). Both retinal glia and neurons are compromised early in the disease progression and display altered metabolic functions and deregulated neurotrophic support. In the retina, glia and neurons closely interact with retinal vasculature to maintain the homeostasis necessary for normal retinal function. Diabetes disturbs the interaction between these cells.

It has been shown that retinal neurodegeneration causes early microvascular changes that occur in diabetic retinopathy. These changes include the breakdown of the blood-retinal barrier (BRB), vasoregression and impairment of neurovascular interaction [6,8–12]. Feng *et al.*, 2009, demonstrated using transgenic mice that neurodegeneration and glial activation initiate vasoregression [11]. Previously, studies demonstrated that retinal hemodynamic response is regulated by the activity of inner retinal neurons and activation of glial cells [8]. In addition, Kusari and coworkers reported that both memantine and briomidine treatments in animal models of diabetes exhibited neuroprotection in addition to reduced VEGF protein levels and reduced blood retinal barrier breakdown [9,10].

In the diabetic retina, apoptosis of neurons and activation of glial cells may cause oxidative stress. Diabetes-induced metabolic dysregulation is considered the major cause of increased oxidative stress, which damages those retinal cells. Several studies have demonstrated that neuronal cells die by apoptosis as the expression of Bax (Bcl-2-associated X protein), a pro-apoptotic protein, is found in increased levels in diabetic retinas [13,14]. In addition, other studies have shown an increased level of terminal nick-end labeling (TUNEL) positive cells correlated with increased levels of caspases (the enzymes involved in apoptosis) in neural retinas of diabetic rats [7]. This confirms the increase in apoptosis within the inner retina in diabetes.

In recent years, treatment options, which have been developed for advanced stages of DR, have inclined towards clinical management of the complications of DR, such as proliferative-DR or macular edema. In addition, much attention has been given to protecting retinal vessels, with less attention given to neuroprotection in DR. Therefore, better understanding of the molecular mechanisms that lead to neurodegeneration is needed. New therapeutic targets are also needed to protect neuronal deaths early in DR. In this review, we discuss recent advances in understanding the neurogeneration in diabetic retina, especially due to metabolic dysregulation, as well as possible neuroprotective strategies.

## 2. Neurodegenerative Factors and Potential Therapeutic Targets

During the past few decades, hyperglycemia has been considered the major contributor and upstream inducer in the progression of DR. However, a number of studies suggest that excess plasma glucose may not account for the range of cellular and functional changes in the progression of DR [15–17]. Several studies indicate that even intensive therapy to control blood glucose has some long-term effects on the risk of DR [18,19]. In addition to high glucose, the dysregulated levels of excitotoxic metabolites, such as glutamate, homocysteine, branched chain amino acids, nutrients, hormones and several other factors, have been found to be implicated in neurodegeneration early in DR [20,21].

## 3. Hyperglycemia

Two clinical trials, Diabetes Control and Complications Trial (DCCT) and the United Kingdom Prospective Diabetes Study (UKPDS), have demonstrated that intensive control of hyperglycemia in both type 1 and type 2 diabetes patients significantly reduced the risk of DR development and progression. Numerous studies have shown that hyperglycemia/diabetes activates several metabolic pathways. These pathways mediate oxidative stress, amplifying retinal tissue damage, which plays an important role in the pathogenesis of diabetic complications, including DR [20,22]. Sources of oxidative stress may include hyperglycemia-induced increased flux through the polyol pathway, depleting NADPH and lowering intracellular levels of the endogenous antioxidant glutathione. This leads to the activation of nuclear factor kappa B (NF-κB), increasing proinflammatory cytokines, chemokines and growth factors, causing further damage to tissue. Other hyperglycemia-induced pathways include activation of protein kinase C (PKC) and increased flux through advanced glycation end products (AGEs) and hexosamine pathways, resulting in increased oxidative stress. Hyperglycemia-induced NADPH oxidase activation, microglial activations and NADH oxidases have also been found to increase oxidative stress in the diabetic retina.

## 4. Glutamate Excitotoxicity

**Scheme 1:**
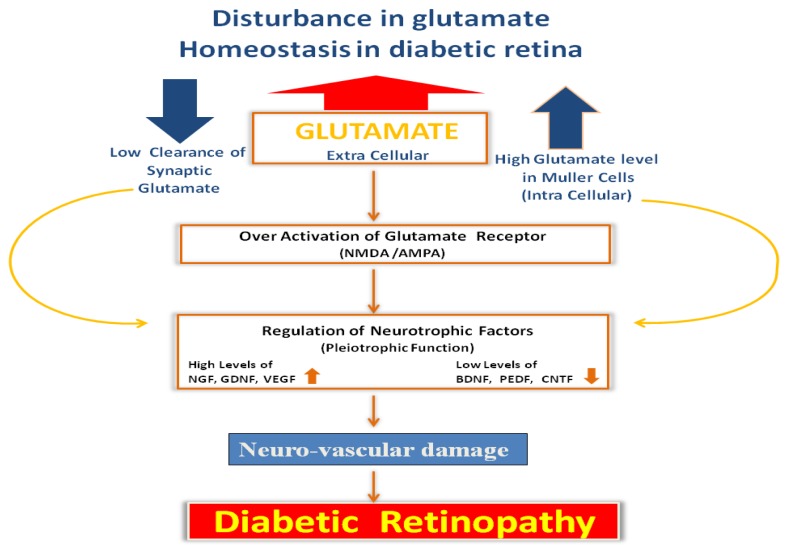
Flow diagram depicting the effect of dysregulated level of glutamate within diabetic retina. Dysregulated levels of glutamate within neurons and glial cells, as well as increased levels in the extracellular space within the retina, can cause excitotoxicity to postsynaptic neurons. That may alter neurotrophic factors, leading to neuro-vascular damage in diabetic retinopathy.

Glutamate is the major excitatory amino acid in the brain and retina. However, numerous studies indicate that due to the disruption of glutamate homeostasis in the diabetic retina, toxic levels of extracellular glutamate in the retina increase, which damage the neurons and initiate the development of DR, as shown in Scheme 1 [23–25]. The major cause of neuronal cell death following glutamate-induced activation of *N*-methyl d-aspartate (NMDA) receptors is the influx of calcium and sodium into cells. This generates free radicals and induces apoptosis [26]. Therefore, strategies to decrease the level of extracellular glutamate or to inhibit the activation of NMDA receptors may decrease neurotoxicity and cell death [25].

Müller cells in the retina are specialized cells, which regulate the level of glutamate both intra- and extra-cellularly within the retina. Müller cells maintain glutamate homeostasis. They immediately take up excess extracellular glutamate released during neurotransmission, thus lowering the excitotoxic level of glutamate. Within Müller cells, glutamate has two metabolic fates: amidation to glutamine and conversion to α-ketoglutarate by transamination. The α-ketoglutarate formed can enter the citric acid cycle (where it is regenerated) or the carbon skeleton of α-ketoglutarate can be irreversibly lost due to the activity of malic enzyme and/or phosphorenol pyruvate carboxykinase and pyruvate kinase. Producing glutamate from the citric acid cycle intermediates requires a nitrogen source. This is provided by excess branched chain amino acids (BCAA), using either of the two isoforms of branched-chain aminotransferase (BCAT): the mitochondrial branched-chain aminotransferase (BCATm) expressed in Müller cells or the cytosolic BCAT isoform (BCATc), which is expressed only in the cytosols of neuronal cells [27,28]. We showed [29] that in the retina, BCATm is expressed exclusively in Müller cell mitochondria, whereas BCATc is expressed in the cytosols of retinal neurons. Gabapentin is a very specific BCATc inhibitor that is approximately 100-fold less active for BCATm. When excised retinas were incubated in a physiological buffer with pyruvate and ^14^CO_2_, gabapentin inhibited ^14^C-glutamate synthesis from ^14^CO_2_ and pyruvate [30,31]. The addition of BCAAs to the medium surrounding the excised retinas increased the oxidation of glutamate to pyruvate, lactate and CO_2_ [27,30]. Therefore, it appears that increased ratios of branched-chain keto acid (BCKA) to BCAA will decrease Müller cell glutamate synthesis and will increase rates of glutamate oxidation [25], whereas decreasing the BCKA/BCAA ratios in diabetic retinas will promote glutamate excitotoxicity by raising the levels of glutamate in Müller cells. It is likely that a high serum BCAA level, which is typical of the diabetic state, is also responsible for diabetic retinopathy, because it may lead to high intra- and extra-cellular glutamate levels in the retina. High levels of BCAA may interfere with glutamate clearance from the synaptic space, thereby increasing the glutamate level and causing excitotoxicity to postsynaptic neurons [25]. Thus, the biochemical mechanism of glutamate excitotoxicity suggests an increase in cell death, resulting in a loss of visual function in diabetes.

The intraocular administration of MK-801, an *N*-methyl d-aspartate (NMDA) receptor antagonist, has been shown to protect against neurodegenerative conditions [32]. Memantine, another NMDA receptor antagonist, demonstrated a neuroprotective effect in retinal ganglion cells (RGCs) when exposed to glutamate [33]. In another study, memantine treatment in animal models of diabetes exhibited neuroprotection in addition to reduced vitreoretinal VEGF protein levels and reduced blood retinal barrier breakdown [9]. Recently, Smith and coworkers showed that a specific sigma receptor-1 ligand, pentazocine, conferred significant neuroprotection in an *in vivo* model of retinal degeneration. This suggests that sigma ligand may be a potential therapy for neurodegenerative diseases of the retina [34]. In a recent study, we found that memantine and gabapentin (Neuritin) administration to diabetic rats reduced caspase-3 activity and reduced the increased levels of ROS in the diabetic retina, suggesting these agents may protect neuronal cells (unpublished data). Thus, these strategies to limit levels of glutamate by regulating glutamate metabolism may be new therapeutic targets in neurodegeneration.

## 5. Nutrients and Vitamins

Homocysteine (Hcys) is a sulfur-containing amino acid. Elevated plasma Hcys has been associated with ocular complications in secondary glaucoma optic atrophy [35], age-related macular degeneration (AMD) and DR [36]. Homocysteine is a by-product of transmethylation reactions and is detoxified by methionine synthetase, which depends on vitamin B12 and folate as coenzymes for its proper function [37]. Determinants of hyperhomocysteinemia, such as low concentrations of folate and B-vitamin coenzymes and altered activities of enzymes involved in the breakdown of homocysteine, are also associated with an increased risk of diabetic complications [38]. Previous studies suggest that impaired activity of the enzyme methylene tetra hydrofolate reductase raises plasma homocysteine levels [39]. Previously, we demonstrated that hyperglycemic and diabetic conditions reduce the expression and activity of the folate transporter and decrease the folate level in the retina, which may have profound implications in diabetes [40]. Folic acid and vitamin B12 supplementation are known to reduce homocysteine levels [41].

More recently, Satyanarayana *et al.* [42] reported an association between vitamin-B12 deficiency and hyperhomocysteinemia in diabetic retinopathy. The effects of elevated homocysteine levels on retinal function in both *in vitro* and *in vivo* models have shown that homocysteine induces apoptosis in RGCs [43,44]. *In vitro* studies of RGC cells and *in vivo* studies in the brain suggest that homocysteine acts as an agonist at the glutamate site of NMDA receptors [45,46]. Other studies have also demonstrated that the neurotoxic effects of homocysteine are also associated with an activation of type II metabotropic glutamate receptors [47,48]. At high millimolar concentrations, homocysteine may also affect glial viability in cultures [49,50]. A case study reported that hyperhomocysteinemia caused by methionine synthase deficiency demonstrated decreased rod response and RGC loss, as determined by ERG and visual evoked potentials [50]. However, less is known about the effects of Hcys on retinal function, including whether homocysteine plays any role in regulating neurotrophic factors. Therefore, strategies to control the level of homocysteine by supplementation of folic acid or vitamin-B12 may be potential treatment strategies to ameliorate neurodegeneration.

## 6. Metabolites of Tryptophan Pathway

Important metabolites in the kynurenine pathway generated by tryptophan degradation are thought to play an important role in neurodegenerative disorders. Glutamate receptor-mediated excitotoxicity and free radical formation have been shown to correlate with decreased levels of the neuroprotective metabolite, kynurenic acid. It has been reported that homocysteine can alter the synthesis of kynurenic acid. In cortical slices and aortic rings, homocysteine stimulates kynurenic acid production at micromolar concentrations and inhibits it when used at millimolar concentrations [51]. It has been suggested that hyperglycemia further increases the harmful effects of elevated homocysteine levels on the availability of kynurenic acid [52]. The kynurenine pathway in diabetes, and especially in DR, has not been studied. A recent study reported elevated levels of kynurenine, kynurenic acid and 3-hydroxykynurenine in the serum of diabetic retinopathy patients [53]. Among the important metabolites of the kynurenine pathway, 3-hydroxykynurenine and quinolinic acid are reported to have neurotoxic effects; however, kynurenic acid is a neuroprotectant [54].

## 7. Neurotrophic Factors

An increasing body of evidence suggests that neuronal guidance molecules, such as neurotrophic factors, play important roles in the interactions between neuronal and vascular cells, thereby regulating the survival, growth and functional maintenance of these cells [55–58]. Dysregulation of neurotrophic factors has been found to cause neurodegeneration and pathologic angiogenesis in diseases, such as diabetic retinopathy, as shown in Scheme 1 [20,57,59,60].

Because the retina is a neuronal tissue, it produces a substantial amount of neurotrophic factors, such as nerve growth factor (NGF), brain-derived neurotrophic factor (BDNF), neurotrophin-3 (NT-3) and NT-4. However, diabetes progressively alters the level of multiple trophic factors/signaling pathways in the retina, reducing the strength of survival signals and increasing apoptosis. Among these neurotrophins, BDNF is produced by neurons and glial cells, which are known to maintain neuronal cells. *In vivo* and *in vitro* studies in brain and neuronal cells suggest that BDNF affects cell differentiation, synaptic connectivity, plasticity, growth and cell survival [58,61,62]. The biological function of BDNF is mediated through high-affinity tyrosine kinase receptors. The effect of BDNF depends mainly on the level and binding affinity to the receptor and the downstream signaling cascades after receptor activation [61]. Rohrer *et al.* [63] showed that the absence of BDNF or its receptor caused serious alterations in retinal function. Furthermore, BDNF has been shown to reduce damage to retinal ganglion cells after optic nerve lesions and to protect neurons in rodents under oxidative stress conditions [64,65]. In addition, BDNF also protects the retina from ischemic injury, promotes the survival of interneurons and plays an important role in the synaptic connections of a large number of neurons [66–68]. More recently, Min and coworkers suggested that BDNF provides a neuroprotective effect by increasing glutamate uptake and the upregulation of glutamine synthetase in Müller cells under hypoxic conditions [69].

Numerous studies have reported decreased BDNF levels in serum from diabetic patients and in diabetic animals. Reduced BDNF was correlated with insulin resistance, reduced glucose and lipid metabolism and increased food consumption [70–72]. In the animal model of diabetes, few studies showed that reduced levels of BDNF in the diabetic retina may damage neurons, thereby leading to neurodegeneration [73,74]. However, the role of BDNF in diabetic retinopathy is not fully understood. Regulating the level of BDNF in the diabetic retina may be a promising therapeutic target to protect neurons.

Several reports have documented increases in NGF levels in serum from patients with insulin-dependent diabetes mellitus and in serum and tears from patients with diabetic neuropathy and retinopathy [55]. The increases in NGF levels positively correlated with the DR stage and other diabetes mellitus (DM) parameters. Several studies have indicated nerve growth factor (NGF) as a contributing factor to neurogenic inflammation and its association with hypoxia [75].

GDNF is a member of the transforming growth factor-β (TGF-β)-related neurotrophic factor family. GDNF promotes photoreceptor survival during retinal degeneration that is mediated by the interaction of neurotrophic factors via receptors in Müller glial cells. These, in turn, release secondary factors that act directly to rescue photoreceptors [76]. Endogenous ciliary neurotrophic factor (CNTF) is upregulated in response to retinal injury and may play an important role in neurodegeneration. Treatment with CNTF in combination with BDNF has been shown to rescue photoreceptors in retinal explants, thus conveying its neuroprotective effects [77].

Another important neurotrophic factor is pigment-epithelium derived factor (PEDF), which offers neuroprotective properties [78]. PEDF protects neurons from glutamate mediated excitotoxicity. The level of PEDF is decreased in diabetic retinopathy. Therefore, enhancing the expression of PEDF can be a therapeutic target to protect neurons.

In addition to the widely known role of VEGF in retinal vascularization, it has also been reported that VEGF is a potential neurotrophic factor. Endogenous VEGF has been found to be involved in the maintenance and function of retinal neurons. It is also a survival factor for photoreceptor cells [79]. A study by Li group has shown that treatment with VEGF protects retinal ganglion cells in various models of neurotoxicity [80]. In another study, inhibition of VEGF in the normal adult retina led to a significant loss of ganglion cells [81]. Therefore, it is important to consider neuroprotection, especially in the case of proliferative diabetic retinopathy treatment using anti-VEGF to regress neovascularization.

Insulin has prosurvival actions in the retina and is considered as an important neurotrophic factor for retinal cells. In insulin-deficient diabetic rats, retinal death increased within a few weeks; and an increase in neuronal apoptosis has been observed in diabetic human eyes [3,82]. The normal retina expresses a highly active basal insulin receptor/Akt signaling pathway. In the streptozotocin-induced diabetic rat model, it has been demonstrated that diabetes progressively impairs the constitutive retinal insulin receptor signaling pathway through Akt kinase and suggests that loss of this survival pathway may contribute to the initial stages of DR [83]. The retinal neurons depend on the activity of the insulin receptor to survive [84,85]. The deletion of these receptors leads to degeneration of inner retinal neurons and photoreceptors [86]. Gardner and coworkers have shown that insulin rescues retinal neurons from cell death in the diabetic rat retina; and intraocular injection of insulin restores insulin receptor activity in diabetic rat retinas [3,83]. The administration of supraphysiological doses of insulin further stimulates insulin receptor/Akt signaling prosurvival pathway [87].

People with diabetes suffer from high blood sugar levels, either because of low insulin production, (insulin being a hormone that regulates cell uptake of glucose) or because cells have become resistant to insulin's action. However, the degree of systemic insulin therapy is limited by the risk of hypoglycemia; therefore, long term local delivery of insulin in the retina is needed to protect against neurodegeneration in DR [88].

## 8. Conclusions

It is now widely acknowledged that diabetic retinopathy is a neurovascular disease. Numerous studies supported neurodegeneration as an early event in the diabetic retina, using a variety of animal model systems. Numerous studies have also investigated mechanisms of neurodegeneration with the aim of uncovering a promising target for successful neuroprotection. However, the exact molecular mechanism of neuronal damage in the diabetic retina is still not known. Diabetes-induced dysregulated levels of excitotoxic metabolites, altered neurotrophic support/signaling and oxidative stress are among the potential causes of neurodegeneration. Increasing interest has been shown in protecting retinal neurons, especially retinal ganglion cells, which are vulnerable to be damaged by diabetes. This can cause vision deficit early in the disease progression, which may implicate it in vascular damage later. A continuous effort is required to better understand the mechanism of diabetes-induced neurodegeneration. That effort may help in discovering a better target and molecule(s) to protect neurons in order to ameliorate the progression of diabetic retinopathy.
